# Subcortical Brain‐Age Gaps Reveal Asymmetric Aging Patterns in Parkinson's Disease With Cognitive Impairment

**DOI:** 10.1002/brb3.71202

**Published:** 2026-01-15

**Authors:** Sadegh Ghaderi, Ali Fathi Jouzdani, Ali Mohammad Pourbagher‐Shahri, Sana Mohammadi

**Affiliations:** ^1^ Neuromuscular Research Center, Department of Neurology, Shariati Hospital Tehran University of Medical Sciences Tehran Iran; ^2^ Department of Neuroscience and Addiction Studies, School of Advanced Technologies in Medicine Tehran University of Medical Sciences Tehran Iran; ^3^ School of Cognitive Sciences Institute For Research in Fundamental Sciences (IPM) Tehran Iran; ^4^ Neuroscience and Neoplasia AI Research Group (NAIRG), Neurophysiology Research Center, Institute of Neuroscience and Cognition, School of Medicine Shahid Beheshti University of Medical Sciences Tehran Iran; ^5^ Neuroscience Research Center Mashhad University of Medical Sciences Mashhad Iran; ^6^ Department of Medical Sciences, School of Medicine Iran University of Medical Sciences Tehran Iran; ^7^ School of Medicine Hamadan University of Medical Sciences Hamadan Iran

**Keywords:** MCI, PD, pathological aging, structural MRI

## Abstract

**Aim:**

The study utilized MRI‐derived brain structure age (BSA) to compare global and regional subcortical BSA among healthy controls (HCs), Parkinson's disease (PD) patients with normal cognition (PD‐NC), and mild cognitive impairment (PD‐MCI), identifying regions with accelerated aging and linking altered BSA to native volumes.

**Methods:**

We analyzed structural MRI data from 55 participants (22 HCs, 18 PD‐NC, 15 PD‐MCI) using the volBrain platform to estimate global and regional subcortical BSA. Group differences in age, global, and regional BSA were tested via Kruskal‐Wallis. Follow‐up analyses included Pearson correlations for significant regions and ANOVAs where assumptions were met.

**Results:**

No significant group differences were found for chronological age (*p* = 0.111) or global BSA (*p* = 0.143). However, at the regional level, non‐parametric analyses revealed significant group differences in the predicted age of the left amygdala (H = 6.42, *p* = 0.040) and the left basal forebrain (H = 6.01, *p* < 0.05), though effect sizes were small (ε^2^ ≤ 0.07). The predicted ages of these two regions were highly collinear (*r* = 0.992). Subsequent parametric tests and Bonferroni‐corrected pairwise comparisons on other subcortical regions did not yield any significant differences.

**Conclusion:**

Accelerated aging appears to be a localized and asymmetric process confined to the limbic‐cholinergic network, specifically involving the left amygdala and basal forebrain. Accelerated brain aging in PD is not global but a localized, asymmetric process in the left limbic‐cholinergic network. Regional brain‐age metrics offer a sensitive biomarker for detecting the specific neurodegeneration linked to cognitive decline.

## Graphical abstract

Accelerated brain aging in Parkinson's disease is not global but a localized, asymmetric process in the left limbic‐cholinergic network. Regional brain‐age metrics offer a sensitive biomarker for detecting the specific neurodegeneration linked to cognitive decline.

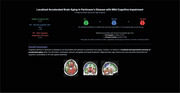

## 1 Introduction

PD is a progressive neurodegenerative disorder characterized by hallmark motor symptoms, including resting tremor, bradykinesia, rigidity, and postural instability, resulting from the loss of dopaminergic neurons in the substantia nigra pars compacta (Kouli, Torsney, and Kuan 2018; Mohammadi et al. 2025; Zhou, Yi et al. 2023). In addition, PD encompasses a broad spectrum of non‐motor symptoms, such as cognitive impairment, psychiatric disorders, sleep disturbances, and autonomic dysfunction, reflecting widespread neuropath logical involvement across multiple neurotransmitter systems and brain regions (Lee and Koh 2015; Schapira, Chaudhuri, and Jenner 2017). Epidemiological studies indicate that PD affects approximately 1–2% of individuals over 60 years of age, with prevalence rising markedly with advancing age (Tysnes and Storstein 2017; Grotewold and Albin 2024). The disease substantially diminishes quality of life, increases dependence on caregivers, and imposes considerable economic burdens on healthcare systems (Luo, Qiao et al. 2024).

When discussing aging, it is important to differentiate three concepts: (a) chronological age, defined as the number of years elapsed since birth; (b) biological age, which reflects the functional state of the body or brain, estimated using biomarkers, and which may progress faster or slower than chronological age (Li, Zhang et al. 2023); and (c) Pathological aging denotes an accelerated, maladaptive progression of disease‐related processes that lead to premature structural and functional deterioration (López‐Otín, Blasco et al. 2013; López‐Otín, Blasco et al. 2023). Age is one of the strongest risk factors for PD, with substantial overlap in the pathogenic mechanisms underlying biological aging and disease progression (Reeve, Simcox, and Turnbull 2014). In major neurodegenerative disorders such as Alzheimer's disease (AD), amyotrophic lateral sclerosis (ALS), and PD, pathological aging represents an accelerated or atypical aging process, most notably associated with disease states (Guo, Huang et al. 2022; Jin and Cai 2023; Mohammadi, Ghaderi et al. 2025). Healthy aging is characterized by the gradual accumulation of oxidative stress (Mossad, Batut et al. 2022), mitochondrial inefficiency (Tracy, Madero‐Pérez et al. 2022), and mild disruptions in proteostasis, including autophagy and lysosomal degradation (Nixon 2020, Aman, Schmauck‐Medina et al. 2021), leading to region‐specific cortical atrophy, white‐matter loss, and subtle cognitive slowing (Blinkouskaya, Caçoilo et al. 2021). Concurrently, declining autophagy and lysosomal function impair the degradation of misfolded proteins, initiating a pathological vicious cycle that accelerates dopaminergic neuron loss, a hallmark of PD (Hou, Watzlawik et al. 2020). In pathological aging, these mechanisms are markedly intensified: excessive production of reactive oxygen species (ROS) induces widespread macromolecular damage (Giorgi, Marchi et al. 2018); age‐related mitochondrial failure becomes catastrophic in high‐energy–demand neurons such as those in the substantia nigra (Reeve, Simcox, and Turnbull 2014); and impaired protein clearance permits the accumulation of toxic aggregates (like misfolded α‐synuclein), promoting Lewy body formation and neuroinflammation (Mahul‐Mellier, Burtscher et al. 2020). Neuroinflammation, driven by reactive glial cells and pro‐inflammatory cytokines, further exacerbates neuronal damage and fosters additional protein aggregation (Zhang, Xiao et al. 2023).

Human brain growth and aging are region‐dependent, non‐linear, and consist of synchronized periods of growth and atrophy of variable magnitude and timing across regions (Nguyen, Clément et al. 2024). In healthy aging, brain volume loss occurs in a progressive, region‐specific pattern: the frontal lobe undergoes substantial shrinkage during adulthood, particularly in the dorsolateral prefrontal cortex beginning around age 40; the temporal lobe shows a more modest decline, with the inferior temporal cortex largely preserved; whereas the parietal and occipital lobes remain relatively unchanged (Lemaitre, Goldman et al. 2012). Subregions like the hippocampus experience dendritic regression, synaptic loss, telomere shortening, and diminished neurogenesis (Babcock, Page et al. 2021); the amygdala and entorhinal cortex alone create sparse tangles (Makkinejad, Schneider et al. 2019), while white‐matter integrity is gradually lost, slowing processing speed and multitasking (Rieckmann, Van Dijk et al. 2016). Pathological aging magnifies these changes (López‐Otín, Blasco et al. 2023). In PD, oxidative damage and mitochondrial impairment result in the degeneration of dopaminergic neurons in the substantia nigra, adding to cortical and subcortical deterioration (Henrich, Oertel et al. 2023; Mohammadi, Ghaderi et al. 2025).

PD‐MCI is characterized by clinical, cognitive, and functional criteria and is an intermediate point between normal cognition and dementia in patients with PD (Cammisuli, Cammisuli et al. 2019). Pathological aging accelerates cognitive decline in PD‐MCI by promoting advanced brain aging and structural degeneration, whereas its impact in PD with normal cognition (PD‐NC) is comparatively limited, allowing cognitive function to remain largely preserved (Jellinger 2023). This selective impact indicates aging to be a leading modulator of PD's cognitive trajectory and underscores the need for individualized treatment strategies. Chronological age is a predictor of PD risk, but biological brain age, quantified on MRI “brain‐age” gaps, dopamine scans, and cerebrospinal fluid (CSF) biomarkers, generally surpasses actual years in PD, particularly in the presence of cognitive impairment (Teipel, Hoffmann et al. 2024). Monitoring biological versus chronological age allows earlier diagnosis, tailored interventions, and enhanced monitoring of PD disease progression (Moqri, Herzog et al. 2023). Nowadays, BSA is an emerging biomarker derived from structural magnetic resonance imaging (sMRI) that estimates the whole biological age of the brain and subregions (Nguyen, Clément et al. 2024). Foundational work using deep learning on raw MRI data has established that brain‐predicted age is a highly reliable and heritable phenotype, capable of serving as a robust biomarker for individual differences in the brain aging process (Cole et al. 2017). BSA may reveal accelerated brain aging (pathological aging) compared to chronological age, reflecting disease‐related neurodegeneration (Mohammadi, Ghaderi et al. 2025).

Consequently, our cross‐sectional analysis study aimed to (a) compare global and regional brain‐predicted ages, derived from structural MRI, among HCs, PD‐NC, and PD‐MCI; (b) identify subcortical regions that exhibit significant deviations in biological (brain‐predicted) age across these groups; and (c) examine the relationship between regional brain‐predicted ages and native volumetric measures in the subcortical structures showing group differences.

## 2 Methods

### 2.1 Participants

This study represents a secondary analysis of a publicly available dataset originally reported by Kemp et al. (2025) (Kemp, Eubank et al. 2025). In the original study, individuals with a clinical diagnosis of PD and HCs were enrolled in a two‐year longitudinal investigation of cognitive impairment at the New York University (NYU) Grossman school of medicine. Written informed consent was obtained from all participants prior to study procedures.

A subset of participants elected to undergo optional neuroimaging, with several excluded due to motion artifacts or other scanning issues. The final neuroimaging cohort comprised right‐handed participants categorized into three groups: HCs, PD‐NC, and PD‐MCI. All imaging was conducted at NYU's center for biomedical imaging, de‐identified, and subsequently transferred to the University of Arkansas for medical sciences for processing.

Group classification was based on standardized clinical and cognitive assessments. Disease severity in PD participants was evaluated using the movement disorders society unified PD rating scale (MDS‐UPDRS) (Goetz, Tilley et al. 2008) and the Hoehn and Yahr scale (Hoehn and Yahr 1967). Cognitive performance was assessed with the montreal cognitive assessment (MoCA) (Nasreddine, Phillips et al. 2005) and the repeatable battery for the assessment of neuropsychological status (RBANS) (Randolph, Tierney et al. 1998).

### 2.2 Image Analysis and BSA Measurements

As mentioned earlier, all imaging was conducted at a single site (NYU's center for biomedical imaging) on a Siemens 3T trio MRI scanner, ensuring a consistent acquisition environment across all groups. Preprocessing began with voxel resampling to 1 × 1 × 1 mm^3^ using FreeSurfer's mri_convert command (https://surfer.nmr.mgh.harvard.edu/fswiki/mri_convert), ensuring uniform spatial resolution across scans. This isotropic standardization was required for subsequent BSA analysis (Denis de Senneville, Manjón, and Coupé 2020; Nguyen, Clément et al. 2024) performed with the volBrain platform (https://volbrain.net/services/BrainStructureAges).

**FIGURE 1:**
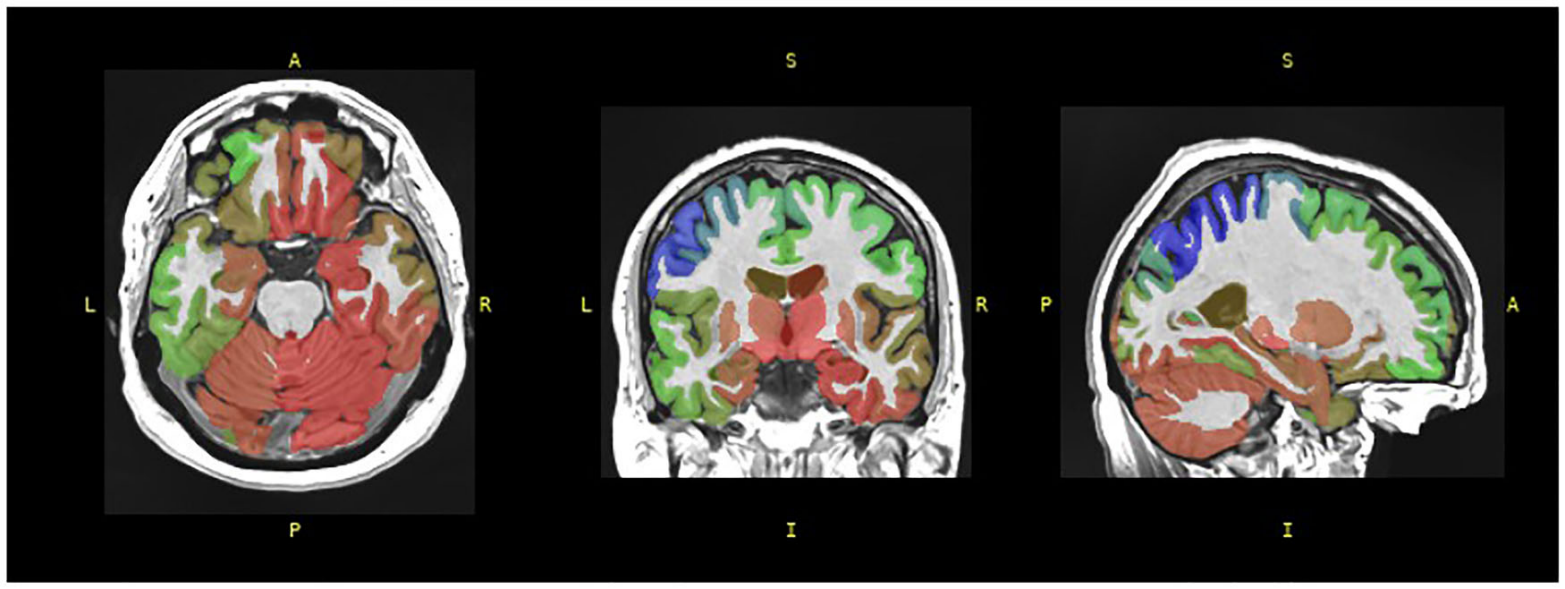
A structural analysis of T1‐weighted MRI was conducted using the volBrain platform to estimate (BSA. The estimated age range is expressed in years, with the chronological age of the PD patient recorded as 63 years.

BSA estimation was conducted using volBrain (Manjón and Coupé 2016), a cloud‐based system that applies deep learning models trained on a normative cohort spanning 0–100 years (Figure 1). The pipeline automatically extracts anatomical features from cortical and subcortical structures to generate region‐specific biological age estimates, which are then aggregated into a global BSA score.

For neuroanatomical segmentation, AssemblyNet was employed to parcellate brain structures into anatomically defined regions (Coupé, Mansencal et al. 2020). The following subcortical structures were volumetrically quantified: accumbens, amygdala, basal forebrain, caudate, hippocampus, pallidum, putamen, and thalamus. All processed imaging data and derived analyses are available upon request from the corresponding authors.

### 2.3 Statistical Analyses

All statistical analyses were performed using SPSS 27.0 (IBM Corp.), with a significance level of *p* < 0.05 established for all two‐tailed tests. Given the non‐normal distribution of some variables, demographic, clinical, and cognitive data were compared across the three cohorts (HCs, PD‐NC, and PD‐MCI) using non‐parametric methods: Kruskal‐Wallis H tests for continuous variables and chi‐square tests for categorical variables. This non‐parametric approach was also extended to the primary analysis of imaging data, where Kruskal‐Wallis tests were used to compare chronological age, global BSA, and all regional subcortical age estimates across the groups. The effect size for these analyses was quantified using epsilon‐squared (ε^2^). In instances where a significant omnibus effect was observed, post‐hoc pairwise comparisons were conducted with Bonferroni correction to control the family‐wise error rate.

Following the initial group comparisons, targeted follow‐up analyses were conducted. For the specific brain regions that demonstrated a significant group difference in predicted age based on the Kruskal‐Wallis test, Pearson correlation coefficients (r) were computed to assess the association between the predicted age estimates and their corresponding native volumetric measures. A secondary parametric analysis was also performed for the four regional age estimates that met the assumptions of normality and homoscedasticity. For these specific regions, one‐way analyses of variance (ANOVAs) were used to re‐examine group differences. The effect sizes for these parametric tests were reported as both eta‐squared (η^2^) and omega‐squared (ω^2^), including their 95% confidence intervals. All subsequent pairwise group comparisons for the ANOVA results were adjusted for multiple comparisons using the Bonferroni method to ensure statistical rigor.

## 3 Results

### 3.1 Overview of Subject Characteristics

**FIGURE 2:**
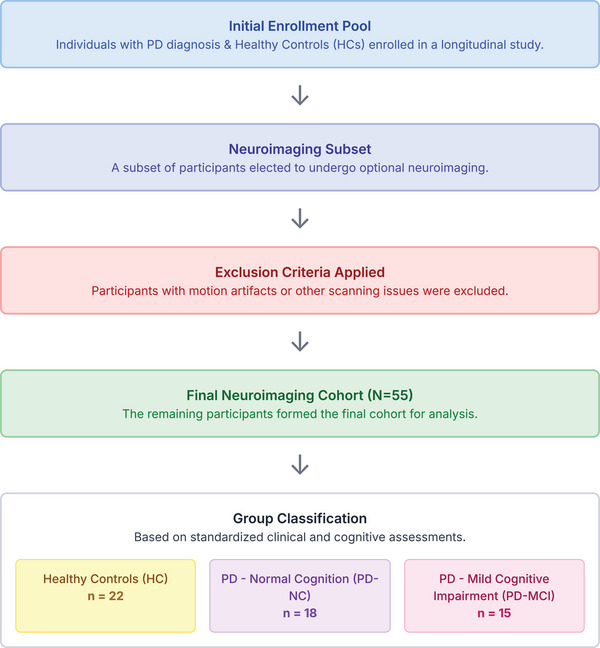
Illustrates the sequential process through which the initial participant pool was systematically refined and subsequently classified into three distinct groups, HCs, PD‐NC, and PD with PD‐MCI, for the purpose of analysis.

We analyzed the cross‐sectional MRI data of 55 participants selected for the final analysis in the Kemp et al. (2025) study (Kemp et al. 2025). The final analytic cohort comprised 22 HCs, 18 patients with PD‐NC, and 15 patients with PD‐MCI (Figure 2). The proportion of males was lower in HCs (32 %) than in both PD cohorts (PD‑NC 61 %, PD‑MCI 73 %; *p* < 0.05).

The mean age differed across groups, with PD‐NC individuals being approximately 5 years younger than those in the HCs group (65.9 ± 5.2 vs. 70.7 ± 7.5 years; *p* < 0.05). But the PD‐MCI group's mean age (69.9 ± 9.3 years) was similar to the HCs group. Participants with PD, with or without cognitive impairment, had more years of education than controls (PD‑NC 17.8 ± 2.3, PD‑MCI 18.1 ± 1.5, HCs 16.3 ± 1.6 years; both *p* < 0.05). PD severity assessments were similar between the two PD groups, as disease duration (7.1 ± 4.4 vs 7.5 ± 5.9 years), MDS‑UPDRS motor scores (24.8 ± 10.9 vs 29.7 ± 9.5), and Hoehn‐Yahr stage (both 2.4 ± 0.6) were not significantly different between the two PD groups.

Cognitive function assessments were significantly different as the PD‑MCI group had a lower score than both the HC and the PD‑NC groups on the MoCA (22.7 ± 3.5 vs 26.0 ± 2.3 and 27.1 ± 1.9, respectively; *p* < 0.05) as well as the repeatable RBANS global index (82.8 ± 6.8 vs 107.9 ± 10.1 and 101.6 ± 16.1; *p* < 0.0001). These patterns replicate the demographic and neuropsychological profile reported in the original dataset by Kemp et al. (2025) (Kemp et al. 2025), on which the current imaging analyses are based (Table 1).

**TABLE 1:** Demographics, PD duration, measures of PD severity (MDS‐UPDRS, Hoehn‐Yahr stage), and general cognitive function assessment scores (MoCA and battery for the assessment of neuropsychological status (RBANS)).

—	—	Healthy controls (HCs, *n* = 22)	PD without cognitive impairment (PD‐NC, *n* = 18)	PD with mild cognitive impairment (PD‐MCI, *n* = 15)	Significant differences
—	Numbers (sex: M/F)	22(7/15)	18(11/7)	15(11/4)	%M: HC < PD‐NC, PD‐MCI *
—	Mean age ± SD	70.7 ± 7.5	65.9 ± 5.2	69.9 ± 9.3	PD‐NC < HC *
—	Years of Edu. ± SD	16.3 ± 1.6	17.8 ± 2.3	18.1 ± 1.5	HC < PD‐NC, PD‐MCI *
—	PD duration ± SD	NA	7.1 ± 4.4	7.5 ± 5.9	(not significant)
PD severity	MDS‐UPDRS ± SD	NA	24.8 ± 10.9	29.7 ± 9.5	(not significant)
	Hoehn‐Yahr ± SD	NA	2.4 + 0.6	2.4 + 0.6	(not significant)
Cognitive function assessment	MoCA ± SD	26.0 ± 2.3	27.1 ± 1.9	22.7 ± 3.5	PD‐MCI *<* HC, PD‐ NCI*
	RBANS ± SD	107.9 ± 10.1	101.6 ± 16.1	82.8 ± 6.8	PD‐MCI *<* HC, PD‐NC †

*Note*: * *p* < 0.05; †*p* < 0.0001; SD = standard deviation; NA = not applicable.

### 3.2 Brain‐Predicted Age and Regional Subcortical Measures

Table 2 summarizes the chronological age, global BSA prediction, and regional subcortical age estimates, as well as their non‐parametric group comparisons. Chronological age did not differ significantly across groups (H = 4.402, *p* = 0.111). Similarly, the global BSA showed no group effect either (H = 3.887, *p* = 0.143).

**TABLE 2:** Age prediction and volumetry results for subcortical structures across the HCs, PD‐NC, and PD with PD‐MCI groups.

—	—	—	HC	PD‐NC	PD‐MCI	Kruskal–Wallis results		
—		—	Median [percentile 25‐percentile 75]			H	*P*	ε^2^ (a)
Age (Years)	Chronological		71 [66–75]	65 [63–67]	67 [63–75]	4.402	0.111	0.05
	Biological prediction		71.28 [65.93–75.13]	67.41 [64.73–72.18]	73.57 [70.73–76.45]	3.887	0.143	0.04
	Accumbens	R	72.02 [63.91–75.22]	73.90 [70.40–75.98]	73.90 [70.40–75.98]	4.571	0.102	0.00
		L	71.80 [65.43–74.63]	73.43 [70.84–77.44]	73.43 [70.84–77.44]	4.616	0.099	0.01
	Amygdala	R	71.58 [64.92–76.17]	73.52 [71.63–77.28]	73.52 [71.63–77.28]	4.693	0.960	0.02
		L	71.11 [64.56–75.09]	74.47 [70.72–77.47]	74.47 [70.72–77.47]	6.422	0.040	0.07
	Basal forebrain	R	71.84 [65.01–75.60]	74.34 [71.24–76.24]	74.34 [71.24–76.24]	4.439	0.109	0.03
		L	71.36 [65.44–74.72]	74.48 [71.08–78.18]	74.48 [71.08–78.18]	6.009	0.05	0.04
	Caudate	R	71.85 [64.61–75.01]	72.70 [70.41–76.13]	72.70 [70.41–76.13]	3.761	0.152	0.02
		L	72.19 [65.42–74.05]	72.00 [69.69–77.63]	72.00 [69.69–77.63]	4.081	0.130	0.02
	Hippocampus	R	71.60 [65.00–74.96]	73.29 [69.10–77.95]	73.29 [69.10–77.95]	—	—	—
		L	70.75 [64.42–76.18]	74.20 [69.25–77.28]	74.20 [69.25–77.28]	—	—	—
	Pallidum	R	71.84 [64.24–75.33]	74.94 [69.80–75.81]	74.94 [69.80–75.81]	4.330	0.115	0.05
		L	72.13 [65.01–74.85]	73.91 [69.39–77.85]	73.91 [69.39–77.85]	5.250	0.072	0.06
	Putamen	R	71.81 [65.50–75.53]	74.82 [69.65–75.97]	74.82 [69.65–75.97]	—	—	—
		L	72.32 [65.80–75.05]	75.04 [69.14–78.20]	75.04 [69.14–78.20]	5.702	0.058	0.07
	Thalamus	R	70.96 [63.80–75.16]	75.09 [69.04–75.92]	75.09 [69.04–75.92]	—	—	—
		L	71.31 [63.57–76.13]	73.03 [68.62–77.15]	73.03 [68.62–77.15]	5.298	0.071	0.06
Volume (cm^3^)	Accumbens	R	0.35 [0.28–0.43]	0.39 [0.36–0.43]	0.35 [0.25–0.39]	—	—	—
		L	0.38 [0.31–0.50]	0.48 [0.41–0.55]	0.41 [0.34–0.48]	—	—	—
	Amygdala	R	1.04 [0.95–1.11]	1.18 [1.00–1.30]	1.09 [0.98–1.22]	—	—	—
		L	1.02 [0.91–1.05]	1.11 [0.94–1.18]	1.02 [0.89–1.15]	—	—	—
	Basal Forebrain	R	0.33 [0.30–0.37]	0.32 [0.31–0.39]	0.37 [0.33–0.41]	—	—	—
		L	0.39 [0.36–0.46]	0.39 [0.35–0.46]	0.45 [0.35–0.48]	—	—	—
	Caudate	R	2.67 [2.41–3.00]	2.85 [2.44–3.08]	2.68 [2.55–2.74]	—	—	—
		L	2.70 [2.45–3.01]	2.90 [2.37–3.04]	2.68 [2.54–2.80]	—	—	—
	Hippocampus	R	3.35 [3.14–3.57]	3.76 [3.51–4.00]	3.58 [3.25–3.75]	—	—	—
		L	3.28 [3.07–3.59]	3.70 [3.21–4.07]	3.54 [3.07–3.71]	—	—	—
	Pallidum	R	1.23 [1.11–1.39]	1.40 [1.26–1.57]	1.37 [1.14–1.42]	—	—	—
		L	1.32 [1.21–1.43]	1.50 [1.41–1.66]	1.42 [1.27–1.58]	—	—	—
	Putamen	R	3.76 [3.55–4.16]	4.42 [3.74–4.75]	4.19 [3.73–4.51]	—	—	—
		L	3.84 [3.54–4.24]	4.52 [3.85–4.70]	4.16 [3.76–4.48]	—	—	—
	Thalamus	R	7.33 [6.97–8.02]	8.19 [7.72–8.74]	7.93 [7.11–8.23]	—	—	—
		L	7.19 [6.78–7.64]	7.96 [7.48–8.61]	7.46 [6.79–8.20]	—	—	—
	Subcortical GM (total)	—	40.21 [37.71–43.45]	44.72 [42.35–48.72]	41.94 [39.08–45.38]	—	—	—

Abbreviations; HC  =  healthy controls; PD‑NC  =  Parkinson's disease with normal cognition; PD‑MCI  =  Parkinson's disease with mild cognitive impairment; values are median [25th–75th percentile]. H  =  Kruskal–Wallis statistic, df  =  2; *p*‑values two‑tailed and unadjusted for multiple comparisons; ε^2^ = effect‑size analogue. Magnitude interpretation for ε^2^: small ≈ 0.01, medium = 0.06, large = 0.14.

**FIGURE 3:**
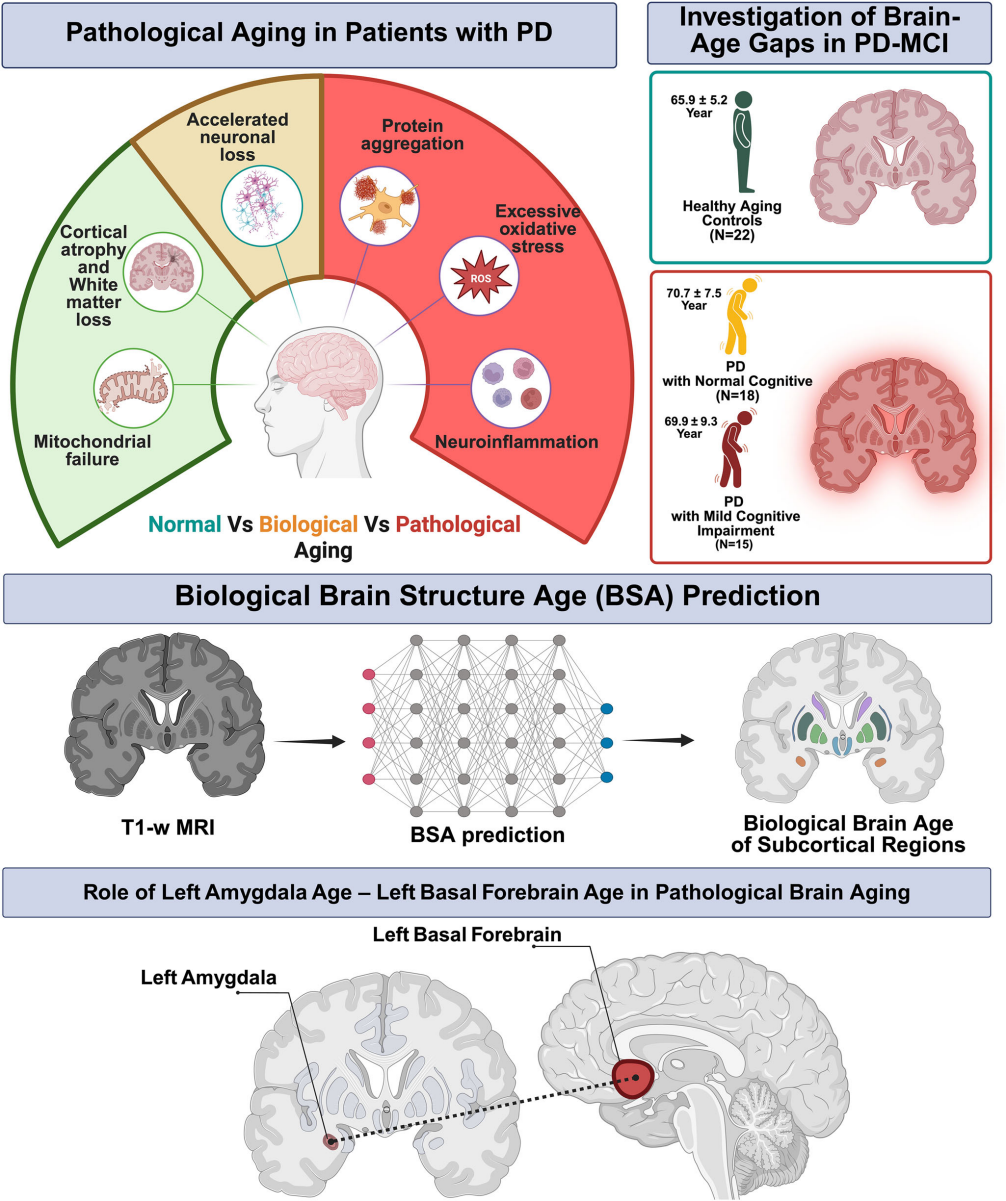
Illustrates the mechanisms of pathological aging, including neuronal loss, cortical atrophy, mitochondrial failure, protein aggregation, oxidative stress, and neuroinflammation, highlighting the contrast between normal, and pathological aging. Significant accelerated aging was localized to the left amygdala and left basal forebrain, key components of the limbic‐cholinergic network, suggesting that regional rather than global brain‐age markers may underlie cognitive decline in PD.

At the regional level, non‐parametric analyses revealed significant group differences in the predicted age of the left amygdala (H = 6.42, *p* = 0.040) and the left basal forebrain (H = 6.01, *p* < 0.05). Examination of median values (Table 2) indicates that this difference was driven by higher predicted brain ages in both PD groups (PD‐NC and PD‐MCI) compared to HCs, suggesting that the accelerated aging effect in these regions is associated with the presence of PD rather than cognitive status specifically (Figure 3). The right‐sided homologues and the remaining regions were nonsignificant. The small ε^2^ values (<0.08) indicate that group membership explained 8 % of the variance in any regional metric. The structural volumetric data measures were collected for subcortical gray matter structures (Table 2). Where differentiation in BSA was observed across regional subcortical ages, volumetric data were used to perform association analyses with the corresponding regional subcortical age estimates (Table 3).

**TABLE 3:** Pearson correlations among left‐hemisphere amygdala and basal forebrain measures (*N * =  55).

Pair (variable 1–variable 2)	*r*	*p* (two‐tailed)	95 % CI (Lower–Upper)
Left Amygdala Age—Left Amygdala vol	−0.211	0.122	−0.449 – 0.060
Left Amygdala Age—Left Basal Forebrain Age	0.992**	<0.001	—
Left Amygdala vol—Left Basal Forebrain vol	0.378**	0.004	—
Left Basal Forebrain Age—Left Basal Forebrain vol	0.411**	0.002	—

*Note*: *r* = Pearson correlation coefficient; two‐tailed significance; ** *p* < 0.01. Predicted ages were obtained with volBrain brain structure ages; native volumes (vol) are raw structure volumes in cm^3^.

Table 3 shows the zero‐order Pearson correlations (*N* = 55) among the predicted age estimates and native volumes of the left amygdala and left basal forebrain. The predicted age of the two regions was collinear (*r* = 0.992, *p* < 0.001), confirming the high redundancy observed across subcortical age metrics in the source dataset. Predicted age was moderately related to the corresponding native volumes (basal forebrain age with basal forebrain volume: *r* = 0.411, *p* = 0.002). On the other hand, native volumes themselves showed only a modest association (*r* = 0.378, *p* = 0.004). No significant correlation emerged between amygdala volume and its own predicted age (*r* = −0.211, *p* = 0.122).

### 3.3 Parametric Group Comparisons of Regional Brain‐Predicted Age (One‐Way ANOVA)

Table 4 presents the four subcortical regions whose age estimates satisfied normality and homoscedasticity assumptions required for parametric testing. In consistency with the previous results, none of the omnibus F‑tests reached the conventional significance threshold (All *p* > 0.05). Left hippocampus showed the largest effect (η^2^ = 0.107), but the group factor still accounted for less than 11 % of the variance, and the p‑value remained slightly above 0.05. All fixed‑effect ω^2^ estimates were close to zero (−0.038 to 0.072), showing a nonsignificant practical impact.

**TABLE 4:** One‑way ANOVA results for regional brain‑age measures.

Region	F (2, 52)	*p*	η^2^	95 % CI η^2^	ε^2^	ω^2^(fixed)	95 % CI ω^2^
Right hippocampus	1.82	0.173	0.065	0.000–0.200	0.029	0.029	−0.038–0.166
Left hippocampus	3.13	0.052	0.107	0.000–0.257	0.073	0.072	−0.038–0.225
Right putamen	1.79	0.176	0.065	0.000–0.198	0.029	0.028	−0.038–0.165
Right thalamus	1.92	0.157	0.069	0.000–0.205	0.033	0.032	−0.038–0.171

Abbreviations: df = degrees of freedom; F = Fisher's F ratio; *p* = p‐value; η^2^ = eta‐squared; ε^2^ = epsilon‐squared; ω^2^ = omega‐squared; CI = Confidence Interval. *Notes*: *p*‐values were derived from one‐way Analysis of Variance (ANOVA) tests applied to regional age estimates that met the assumptions of normality and homoscedasticity. Effect sizes are reported as η^2^ and ω^2^ (fixed effect), with ε^2^ provided for comparison with non‐parametric results. The threshold for statistical significance is set at p < 0.05.

### 3.4 Pair‑Wise Group Comparisons (Bonferroni‐Adjusted)

Table 5 reports the Bonferroni‑corrected pair‑wise contrasts that were measured for the four regions analyzed in the one‑way ANOVA test. Similarly, there were no significant differences between the four regions (adjusted p range 0.064–1.000). The largest numerical gap was observed for the left hippocampus between PD‑NC and PD‑MCI (Mean Diff. = −5.67 years, 95 % CI −11.58 to 0.24, *p* adj = 0.064), but still below the significance criterion after correction. All other contrasts showed smaller mean differences (≤ 4.3 years), wide confidence intervals crossing zero, and no reliable group separation for predicted subcortical age.

**TABLE 5:** Bonferroni‐adjusted pair‐wise comparisons for regional brain‐age measures.

Region	Contrast (I—J)	Mean diff.	Std. error	*p* adj	95 % CI (lower–upper)
Right hippocampus	Control—PD‐NC	+1.49	2.01	1.000	−3.48–6.45
	Control—PD‐MCI	−2.69	2.11	0.627	−7.9 –2.54
	PD‐NC—PD‐MCI	−4.17	2.21	0.192	−9.63–1.28
Left hippocampus	Control—PD‐NC	+1.10	2.17	1.000	−4.27–6.47
	Control—PD‐MCI	−4.58	2.29	0.152	−10.23–1.08
	PD‐NC—PD‐MCI	−5.67	2.39	0.064	−11.58–0.24
Right putamen	Control—PD‐NC	+1.43	2.06	1.000	−3.68–6.53
	Control—PD‐MCI	−2.82	2.17	0.599	−8.20–2.55
	PD‐NC—PD‐MCI	−4.25	2.27	0.200	−9.86–1.36
Right thalamus	Control—PD‐NC	+1.29	1.98	1.000	−3.60–6.18
	Control—PD‐MCI	−2.90	2.08	0.511	−8.04–2.25
	PD‐NC—PD‐MCI	−4.19	2.17	0.179	−9.56–1.19

*Notes*: Mean diff. is calculated as (group I−group J); positive values indicate that group I has the older predicted age. *p* adj values are Bonferroni‑adjusted for the three pair‑wise comparisons within each region. 95 % CI represents the adjusted confidence interval for the mean difference.

Abbreviations: HC  =  healthy controls; PD‑NC  =  Parkinson's disease without cognitive impairment; PD‑MCI  =  Parkinson's disease with mild cognitive impairment.

## 4 Discussion

We found no significant differences among groups for either chronological age or global BSA. At a more granular level, non‐parametric analyses revealed statistically significant but small group effects on the predicted age of the left amygdala and left basal forebrain. The predicted ages of these two regions were highly correlated with each other, and only the basal forebrain's predicted age was moderately associated with its native structural volume. Subsequent parametric and post‐hoc analyses on selected subcortical regions that met the necessary assumptions, including the left hippocampus, failed to show any significant group differences after correcting for multiple comparisons. The most notable trend was a non‐significant, five‐year difference in the left hippocampus's predicted age between the PD‐NC and PD‐MCI groups. Overall, despite some localized signals in the left amygdala and basal forebrain, the results indicate a general absence of robust group differences in brain‐predicted age across the subcortical structures examined.

Studying pathological and biological aging in brain MRI of PD patients is essential for improving diagnosis, understanding disease mechanisms, and enhancing clinical outcomes (Higgins‐Chen, Thrush, and Levine 2021; Jin and Cai 2023; Komleva, Shpiliukova et al. 2025). Aging is the strongest known risk factor for PD, with both incidence and prevalence increasing significantly with advancing age (Reeve, Simcox, and Turnbull 2014). MRI‐based brain‐predicted age estimates can detect subtle deviations from normative aging trajectories and offer insights into neurodegeneration and network vulnerability in PD (More, Antonopoulos et al. 2023). In this study, the global BSA did not differ significantly between controls and PD patients (with or without cognitive impairment), despite clear neuropsychological and demographic differences. Accelerated aging, however, was regionally localized: the left amygdala showed a slight but significant biological age increase, and so did the left basal forebrain. Pearson correlation tests of left‐hemisphere measures found near‐perfect collinearity between the two regions predicted ages, but amygdala age was uncorrelated with its native volume. Conversely, moderate positive correlations were observed between amygdala age and basal forebrain volume, basal forebrain age and volume, and between the two structure volumes. These results suggest redundancy of subcortical measures of age while underscoring the added value of volumetric measurement, and suggest the amygdala and basal forebrain may share similar pathological changes in PD (Harding, Stimson et al. 2002; Rosenberg‐Katz, Herman et al. 2016; Blair, Barrett et al. 2019; Charroud and Turella 2021).

Despite clear neuropsychological and demographic differences between the three groups in this study, our results showed similar chronological and global BSA between them. This contrasts with previous studies in PD patients, which have reported global BSA exceeding chronological age by about three years, detectable even at diagnosis and associated with longer disease duration and greater clinical impairment (Eickhoff, Hoffstaedter et al. 2021).

In our sample, the absence of a global age gap may be driven by the significantly higher educational attainment in the PD groups compared with the HCs. This aligns with the cognitive reserve hypothesis, where higher education may buffer against global structural volume loss despite the presence of pathology (Steffener, Habeck et al. 2016). Future studies with matched educational cohorts are required to confirm whether this preservation is a true disease characteristic or a demographic artifact. An alternative reason might be that global brain‐age metrics are less sensitive to region‐specific neurodegeneration, evidence common in PD, since accelerated aging in PD is often limited to cortical, limbic, and subcortical regions instead of being uniform across the brain (Chen, Kuo et al. 2024; Teipel, Hoffmann et al. 2024). This suggests that global brain age measurements might not detect early or localized neurodegenerative changes in PD.

At the regional level, we observed a subtle, left‐lateralized pattern of pathological aging, which was centered on the limbic cholinergic axis, involving the amygdala and basal forebrain. Both regions showed a small but significant increase in biological age with no significant group differences in their native volumes. This supports the interpretation that the observed age effects are not due to age‐related gross atrophy, as this atrophy is driven by synaptic loss, dendritic regression, and moderate neuronal shrinkage. Meanwhile, PD‐related atrophy involves massive neuronal loss and pathological protein aggregation. In addition, PD involves dopaminergic nigrostriatal degeneration and widespread cortical and subcortical atrophy (Watanabe, Senda et al. 2013; Okitsu, Sugaya et al. 2023).

The original rs‐fMRI study of this cohort (Kemp, Eubank et al. 2025) did not identify an analogous network‐specific effect in the limbic cholinergic axis. This suggests that brain‐age modeling may show latent regional vulnerabilities that may go undetected by connectivity analysis. The cholinergic basal forebrain, particularly the nucleus basalis of Meynert (NBM, Ch4) and its posterior subdivision (Ch4p), as well as the amygdala, undergo marked degeneration due to the spread of alpha‐synuclein aggregates, as proposed in the Braak staging hypothesis (Rietdijk, Perez‐Pardo et al. 2017). This is supported by studies showing that both structures exhibit Lewy body formation and neuronal loss (Harding, Stimson et al. 2002; Popescu, Lippa et al. 2004). The basal forebrain provides cholinergic innervation essential for amygdala function; thus, its degeneration may compromise amygdala integrity, contributing to the observed structural correlations (Crimmins, Lingawi et al. 2023; Tuna, Banks et al. 2025).

The amygdala and basal forebrain form part of interconnected neural circuits critical for emotional regulation and cognitive processing (Pessoa 2010; Peck and Salzman 2014). The NBM provides most cortical and amygdalar acetylcholine and supports attention and memory. In PD, α‐synuclein aggregates accumulate in these nuclei. A longitudinal PPMI study found that smaller Ch4p volumes in PD‐MCI predicted cognitive decline over two years, indicating that NBM atrophy in PD is not merely age‐related (Gratwicke and Foltynie 2018). Deformation‐based morphometry has identified a PD‐specific atrophy network encompassing the basal ganglia, basal forebrain, and limbic structures, including the amygdala and hippocampus, with atrophy severity correlating with clinical impairment (Zeighami, Ulla et al. 2015).

The amygdala and basal forebrain are among the earliest limbic regions affected in PD. Amygdala involvement is detectable as early as Braak stage 3, concurrent with substantia nigra pathology, with Lewy bodies and neurites showing nucleus‐specific vulnerability, particularly in the corticomedial, accessory cortical, basolateral, and central amygdalar nuclei (Braak, Braak et al. 1994; Banwinkler, Dzialas et al. 2022; Banwinkler, Theis et al. 2022). These regions form part of a PD‐specific atrophy network identified by deformation‐based morphometry (Zeighami, Ulla et al. 2015). Cross‐sectional MRI consistently demonstrates reduced amygdala volume in PD compared with controls, with more pronounced atrophy in the postural instability/gait difficulty subtype (Rosenberg‐Katz, Herman et al. 2016). The cholinergic basal forebrain, particularly the posterior nucleus basalis of Meynert (Ch4p), is an early and selectively vulnerable target, exhibiting a posterior‐to‐anterior atrophy gradient (Blair, Barrett et al. 2019). Its long, unmyelinated axons and high metabolic demands render it susceptible to oxidative stress, energetic failure, and α‐synuclein–induced toxicity. As the basal forebrain provides dense cholinergic innervation to the amygdala, degeneration within this pathway likely drives synchronized atrophy across both regions, which has been consistently associated with cognitive decline in PD (Blair, Barrett et al. 2019). Specifically, basal forebrain atrophy has been linked to executive dysfunction, whereas amygdala degeneration contributes to impairments in memory and emotional processing, suggesting that their co‐degeneration exacerbates multidomain cognitive impairments characteristic of PD (Pereira, Hall et al. 2020; Labrador‐Espinosa, Silva‐Rodríguez et al. 2023; Slater, Melzer et al. 2024). Taken together, the shared vulnerability of these limbic–cholinergic structures indicates that region‐specific acceleration of biological aging, rather than global brain aging, may underlie early non‐motor symptoms and cognitive decline in PD.

Our findings suggest that global structural aging is preserved in our PD sample, with possible accelerated aging restricted to the left amygdala/basal forebrain complex. The near‐perfect correlation between left amygdala and left basal forebrain ages in our data supports the view that these regions share a common aging signal within the limbic–cholinergic network. This interpretation is further supported by moderate correlations between amygdala age and basal forebrain volume, as well as basal forebrain age and its own volume. These are suggestive of the notion that age‐related change is not simply a by‐product of parallel volumetric loss and may be induced by some other alterations (Björklund and Barker 2024; Bohnen, Roytman et al. 2025; Seo, Oyama, and Yamamoto 2025).

In addition, because the amygdala processes emotion and memory, simultaneous degeneration of the basal forebrain and amygdala probably indicates the breakdown of a network essential for cognitive‐affective integration in PD. Longitudinal MRI data show that Ch4 gray matter density declines at ∼−0.010 units/year, with slightly lower rates in Ch1–3 (Ray, Bradburn et al. 2018). Smaller baseline NBM volumes in early PD predict a ∼3.5‐fold increased risk of developing MCI or dementia and are linked to faster decline in MoCA and memory performance (Blair, Barrett et al. 2019).

The human brain is inherently asymmetric, and PD often exhibits functional lateralization, with motor symptoms typically emerging and progressing unevenly between hemispheres (Heinrichs‐Graham, Santamaria et al. 2017; Lubben, Ensink et al. 2021). Structural MRI studies typically don't show significant left‐to‐right differences in basal forebrain volume. However, functional and metabolic imaging studies reveal subtle hemispheric variations (Blair, Barrett et al. 2019). For example, resting‐state fMRI shows altered BF–cortex connectivity patterns in PD‐MCI, including right‐sided reductions (Zhang, Rong et al. 2023). Regional volumetry has revealed left‐lateralized shrinkage in the cortico‐amygdaloid transition area and a superficial cortex‐like region in patients with PD who have cognitive impairment and hyposmia. This suggests that the left side of the brain is more vulnerable to olfactory‐related limbic structures (Ay, Yıldırım et al. 2023). Other studies, however, found symmetric amygdala volumes, indicating that lateralization may be subregion‐specific rather than global (Filippi, Sarasso et al. 2020). Our findings extend this asymmetry to non‐motor domains, showing accelerated aging confined to the left amygdala and left basal forebrain. This could reflect hemispheric dominance, handedness, or other individual factors, though the mechanisms remain unclear (Wang, Ma et al. 2023).

The sample size was relatively small. Furthermore, the cross‐sectional nature of the data restricts our ability to infer causal relationships or monitor individual trajectories of brain aging over time; future studies would greatly benefit from employing a longitudinal design. Additionally, this study did not include genetic data, which prevented an exploration of how genetic predispositions might impact brain‐predicted age. Moreover, we lacked access to PD severity and cognitive function assessment information, which could have provided further insights.

Significant demographic differences existed between groups, particularly regarding sex and education. While the volBrain pipeline benchmarks against a large normative database, the non‐parametric statistical approach necessitated by our sample distribution precluded the use of covariates (e.g., sex, education, and scanner type) in a regression model. Consequently, we could not statistically isolate the specific variance contributed by these demographic factors from the disease effect. Moreover, while group‐level clinical scores were available, we lacked access to granular, subject‐specific PD severity (UPDRS) and cognitive function scores for the specific subset of participants who underwent imaging. This prevented us from performing direct correlation analyses between BSA and clinical outcomes, which represents a key direction for future translational research.

While the omnibus test for chronological age across the three groups was not significant, pairwise comparisons indicated that the PD‐NC group was younger than the HCs group. Brain‐age prediction models are susceptible to ‘regression to the mean’ bias, where younger subjects may be underestimated and older subjects overestimated. We acknowledge that we did not apply a linear bias correction step; therefore, the specific comparisons involving the PD‐NC group should be interpreted with this potential age‐related confound in mind.

It is important to interpret the near‐perfect correlation (*r* = 0.992) between the predicted ages of the left amygdala and left basal forebrain with caution. While this may reflect a genuine, synchronized biological degeneration of the limbic‐cholinergic network, it also raises the possibility of segmentation artifacts. Given the anatomical proximity of these structures, automated segmentation pipelines may struggle to resolve the precise boundary between the amygdala and the basal forebrain extension, potentially leading to voxel overlap. Future studies utilizing high‐resolution probabilistic maps (Zaborszky et al. 2008) are recommended to validate whether this collinearity is purely biological or partially methodological.

## 5 Conclusions

This study indicates that global BSA does not distinguish between PD‐MCI and PD‐NC, but regional metrics offer greater specificity. We identified localized accelerated aging in the left amygdala and left basal forebrain, regions tied to the limbic, cholinergic network and early α‐synuclein pathology. The strong correlation between predicted ages of these regions suggests a shared aging mechanism. While additional regional effects lacked statistical significance, our findings imply that cognitive decline in PD stems from accelerated aging in vulnerable subcortical structures rather than widespread changes. Global BSA remains preserved, showing no difference between chronological and predicted brain age in patients and controls. Notably, MRI‐derived regional brain‐age metrics emerge as more sensitive biomarkers for detecting early cognitive vulnerability and tracking PD progression, offering valuable insights for diagnosis and monitoring.

## Author Contributions

**Sadegh Ghaderi**: conceptualization, methodology/study design, data curation, writing – original draft preparation, visualization, investigation, supervision, software, formal analysis, validation, writing, reviewing, and editing. **Sana Mohammadi**: writing – original draft preparation, investigation, software, formal analysis, validation, writing, reviewing, and editing. **Ali Fathi Jouzdani**: writing – original draft preparation, investigation, software, formal analysis, validation, writing, reviewing, and editing. **Ali Mohammad Pourbagher – Shahri**: writing – original draft preparation, investigation, software, formal analysis, validation, writing, reviewing, and editing.

## Funding

The authors have nothing to report.

## Ethics Statement

Informed consent was obtained from all individual participants included in the study.

## Conflicts of Interest

The authors declare no conflicts of interest.

## Data Availability

The data that support the findings of this study are openly available in OpenNeuro at https://doi.org/10.18112/openneuro.ds005892.v1.0.0 reference number ds005892. This article contains all the data produced or analyzed during this investigation. Further inquiries should be forwarded to the corresponding author.
